# Chromatin dynamics through mouse preimplantation development revealed by single molecule localisation microscopy

**DOI:** 10.1242/bio.059401

**Published:** 2022-07-29

**Authors:** Marta Portela, Daniel Jimenez-Carretero, Veronica Labrador, Maria Jose Andreu, Elvira Arza, Valeria R. Caiolfa, Miguel Manzanares

**Affiliations:** 1 Centro de Biología Molecular Severo Ochoa, CSIC-UAM, Madrid 28049, Spain; 2 Centro Nacional de Investigaciones Cardiovasculares (CNIC), Madrid 28029, Spain; 3 Bioinformatics Unit, Centro Nacional de Investigaciones Cardiovasculares (CNIC), Madrid 28029, Spain; 4 Microscopy and Dynamic Imaging Unit, Centro Nacional de Investigaciones Cardiovasculares (CNIC), Madrid 28029, Spain; 5 Center for Experimental Imaging, Ospedale San Raffaele, Milan 20132, Italy

**Keywords:** Chromatin, Preimplantation, Super-resolution microscopy, DSTORM

## Abstract

Most studies addressing chromatin behaviour during preimplantation development are based on biochemical assays that lack spatial and cell-specific information, crucial during early development. Here, we describe the changes in chromatin taking place at the transition from totipotency to lineage specification, by using direct stochastical optical reconstruction microscopy (*d*STORM) in whole-mount embryos during the first stages of mouse development. Through the study of two post-translational modifications of Histone 3 related to active and repressed chromatin, H3K4me3 and H3K9me3 respectively, we obtained a time-course of chromatin states, showing spatial differences between cell types, related to their differentiation state. This analysis adds a new layer of information to previous biochemical studies and provides novel insight to current models of chromatin organisation during the first stages of development.

## Abstract (precis)

**SUMMARY:** We have applied super-resolution microscopy to analyse changes in the state of chromatin during the first stages of mouse development, from the two-cell stage to the blastocyst.

## INTRODUCTION

The totipotent mammalian zygote self-organises to generate all embryonic and extraembryonic structures necessary to build a complete organism (Wennekamp et al., 2013). This occurs together with a massive reconfiguration of the chromatin that takes place during the first stages of development (Lu et al., 2016; Ke et al., 2017; Du et al., 2017). These changes allow the transition from extremely differentiated cell types, as are the gametes, to the totipotent embryo, which will shortly engage in a series of early lineage decisions through a gradual loss of cellular potency.

The mouse zygote undergoes successive cleavages, activating transcription at the two-cell stage and establishing the three-dimensional organisation of the chromatin in topologically associated domains (TADs) and compartments at the eight-cell stage (Du et al., 2017; Ke et al., 2017; Flyamer et al., 2017; Collombet et al., 2020). It is around this time when the first differentiation event takes place, leading to the blastocyst stage (32–64 cells) with two cellular populations of different plasticity. These are the multipotent trophectoderm (TE), an extraembryonic population that will generate the placenta, and the pluripotent inner cell mass (ICM) that gives raise to the embryo proper and the extraembryonic yolk sac endoderm (Wolpert et al., 2015). Being able to analyse in detail the dynamics of chromatin through these early stages would allow us to gain further insight into how lineage commitment is occurring. Also, it would provide information on which changes in chromatin dynamics and organisation underlie the transitions in cellular plasticity.

In this context, super-resolution microscopy is of great interest as it gives not only cell-specific but also topologically resolved data. This microscopy field includes techniques that can surpass the light diffraction limit (Abbe, 1873). For example, single molecule localisation microscopy has allowed to image, not only well-known subcellular structures, but also chromatin with a resolution of tens of nanometres. Direct stochastic optical reconstruction microscopy (*d*STORM) (Rust et al., 2006; Van de Linde et al., 2011) is on such method that is based on the accurate localisation of individual fluorescent molecules that are switched on and off. *d*STORM imaging of histones has questioned the 30 nm fibre model defined through electron microscopy (Song et al., 2014), and revealed a heterogeneous grouping of nucleosomes into clutches or nanodomains of 30–50 nm (Fang et al., 2018; Otterstrom et al., 2019; Ricci et al., 2015; Xu et al., 2018). Both live- and fixed-cell super-resolution imaging showed multiple clutches clustered in proximity, forming larger domains in the range of several hundred nanometres (Nozaki et al., 2017). Furthermore, recent studies of epigenetic marks through super-resolution microscopy showed different behaviours at the nanometre scale of heterochromatin in processes of cellular differentiation and carcinogenesis (Xu et al., 2018, 2020). This interesting approach would meet the single cell and topological requirements needed to better understand the first stages of mammalian development.

However, super-resolution techniques have very restrictive physico-chemical requirements (Nahidiazar et al., 2016; Schermelleh et al., 2019), which hinder their application to biological samples different from two-dimensional cultured cells. Here, we have optimised *d*STORM imaging methods for robust and efficient imaging of whole-mount mouse preimplantation embryos, and analysed chromatin dynamics from the two-cell to the blastocyst stage. By following post-translational modifications of Histone 3 linked to active (tri-methylation of lysine 4, H3K4me3) and repressed (tri-methylation of lysine 9, H3K9me3) chromatin, we chart how inactive heterochromatin gradually forms and then decompacts in ICM pluripotent cells. The development of whole-mount super-resolution microscopy in preimplantation embryos will allow obtaining unprecedented detail of sub-cellular processes in the embryo with topological resolution.

## RESULTS

### Whole-mount *d*STORM microscopy with nanometre resolution reveals differences between H3K4me3 and H3K9me3 in mouse blastocysts

*d*STORM achieves its best performance in cultured cells, that offer a two-dimensional thin and transparent substrate for imaging. Other types of samples entail the necessity to adapt both sample preparation and image processing, due to the increase in drift and the abundance of noise and visual artefacts that come from the non-uniform and usually auto-fluorescent background (Deschout et al., 2014). We optimised *d*STORM for imaging in whole-mount mouse preimplantation embryos, being able to acquire super-resolution images of higher-order chromatin structures from spherical objects of approximately 100 µm of diameter and formed by one to four layers of cells. We obtained high quality and reproducible images following two approaches: (1) the adaptation of the mounting protocol, that allowed us to avoid drifting and kept the embryos settled close to the objective, by using β-mercaptoethylamine diluted in glycerol, while retaining a high level of blinking performance (Goossen-Schmidt et al., 2020); and (2) the automation of the homogenisation of the images by global blinking densities, which greatly reduced the variability between samples.

**Fig. 1.**
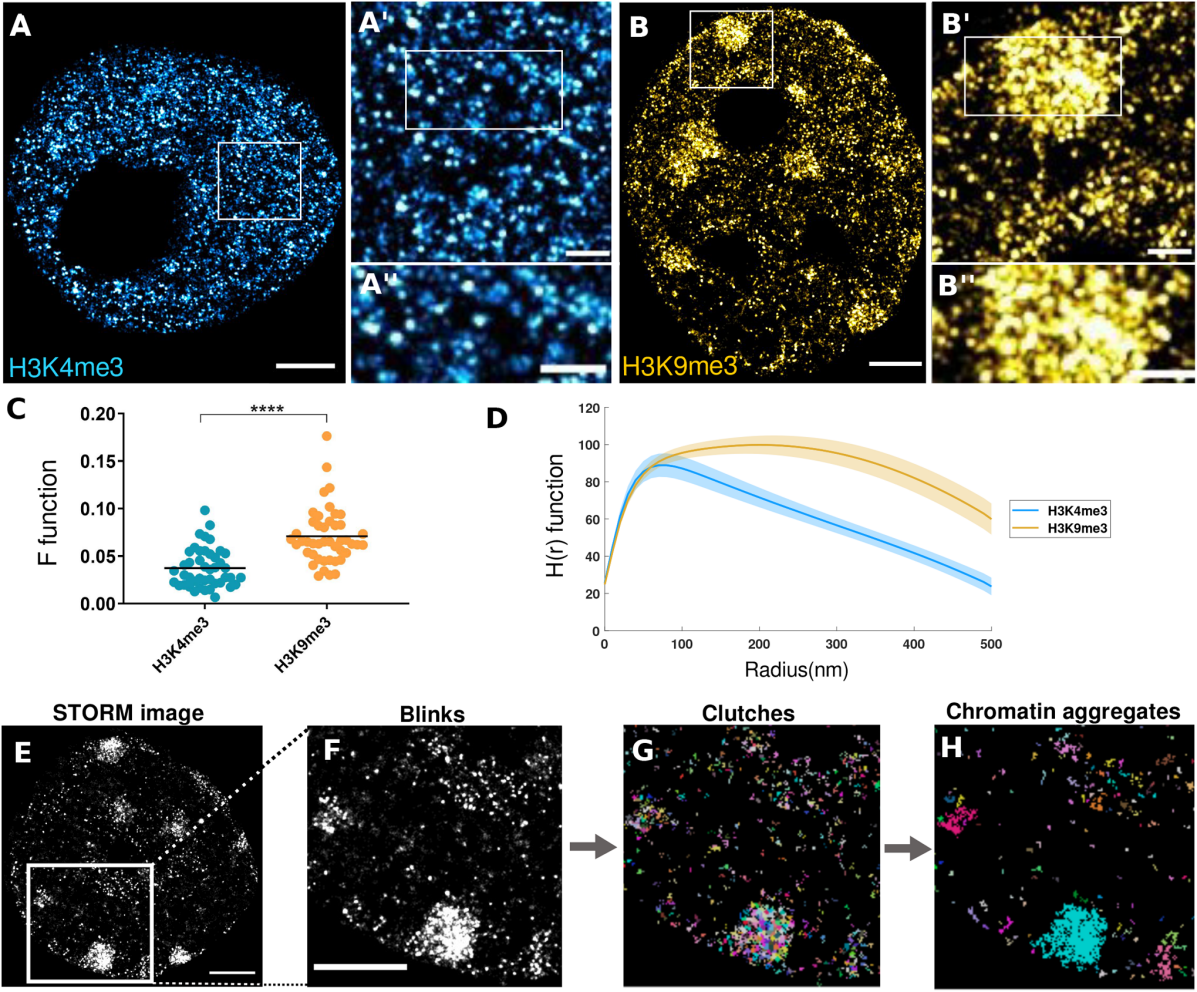
**Super-resolution imaging of H3K4me3 and H3K9me3 in mouse preimplantation embryos.** (A,B) Representative *d*STORM images of trophectoderm nuclei from blastocyst stage embryos stained for H3K4me3 (A) and H3K9me3 (B). Progressively higher magnifications of indicated regions are shown (A′,A″,B′,B″). (C) Values of the F-function for all H3K4me3 (blue) and H3K9me3 (orange) labelled nuclei used in this study, where higher values indicate higher clustering levels. ****, *P*-value<0.0001, *t*-student test. (D) H(r) function or all H3K4me3 (blue) and H3K9me3 (orange) labelled nuclei used in this study, where each function indicates the average amount of molecules that lie at distance r from a reference molecule (mean and standard error of the mean represented with lines and shadows, respectively). (E,F) *d*STORM image of a blastocyst TE nucleus stained with anti-H3K9me3 and a zoom-in of the highlighted region. (G) Segmentation of the region shown in F into nucleosome clutches, shown in different colors. (H) Grouping of clutches into chromatin aggregates based on their proximity and density. Scale bars: 2 µm (A,B,E,F), 500 nm (A′,A″,B′,B″).

To study the chromatin states in preimplantation embryos, we generated super-resolution images of antibody staining for H3K4me3 (that labels transcriptionally active chromatin), and H3K9me3 (that labels transcriptionally repressed chromatin) at different stages of mouse preimplantation development. *d*STORM imaging revealed that both histone marks show a discrete and spatially separated distribution in the nucleus, (Fig. 1A,B), which was not evident with oblique illumination or confocal microscopy (Figs S1A,B and S2C,D) and is in agreement with previous observations in tissue culture (Xu et al., 2018).

H3K4me3 signal showed a punctuated, homogenously distributed pattern throughout the cell nucleus, excluding the nucleoli regions (Fig. 1A–A″). On the other hand, H3K9me3 showed a more heterogeneous distribution, with some highly condensed regions and an accumulation of signal closer to the nuclear and nucleolar periphery (Fig. 1B–B″). These qualitative observations were corroborated studying the F-function of both histone marks by measuring the distance between the function obtained from the signal in our images and randomly distributed dots, indicating higher clustering levels when this distance increases. This analysis showed a significantly higher clustered organisation of H3K9me3, labelling heterochromatin, when nuclei from all stages imaged where analysed together (Fig. 1C). These results were complemented with the study of the H-function for the same set of images (Fig. 1D), where H3K4me3 displays a clear peak defining a low preferential cluster size, while H3K9me3 shows a higher heterogeneity in heterochromatin aggregation sizes.

To gain quantitative insight into the distribution and characteristics of chromatin labelled by different histone marks, we identified different structures based on density and hierarchical clustering methods (Fig. 1E–H). According to the position of blinks in the *d*STORM images (Fig. 1E,F) we defined two main levels of higher-order chromatin organisation. On one hand, nucleosome clutches (Otterstrom et al., 2019; Ricci et al., 2015; Xu et al., 2018), in which single *d*STORM blinks cluster into structures of up to 160 nm diameter, where our data shows a mean radius of ∼40 nm (Fig. 1G and Fig. S1C,D,E). On the other, chromatin aggregates, formed by the clustering of neighbouring clutches based on their proximity into large domains (Fig. 1H).

This approach would allow us to study not only study local interactions of nucleosomes that share the same epigenetic mark, but also analyse the aggregation and compaction dynamics of chromatin in different developmental stages of preimplantation embryos.

### Intranuclear distribution of active and inactive chromatin through preimplantation development

**Fig. 2.**
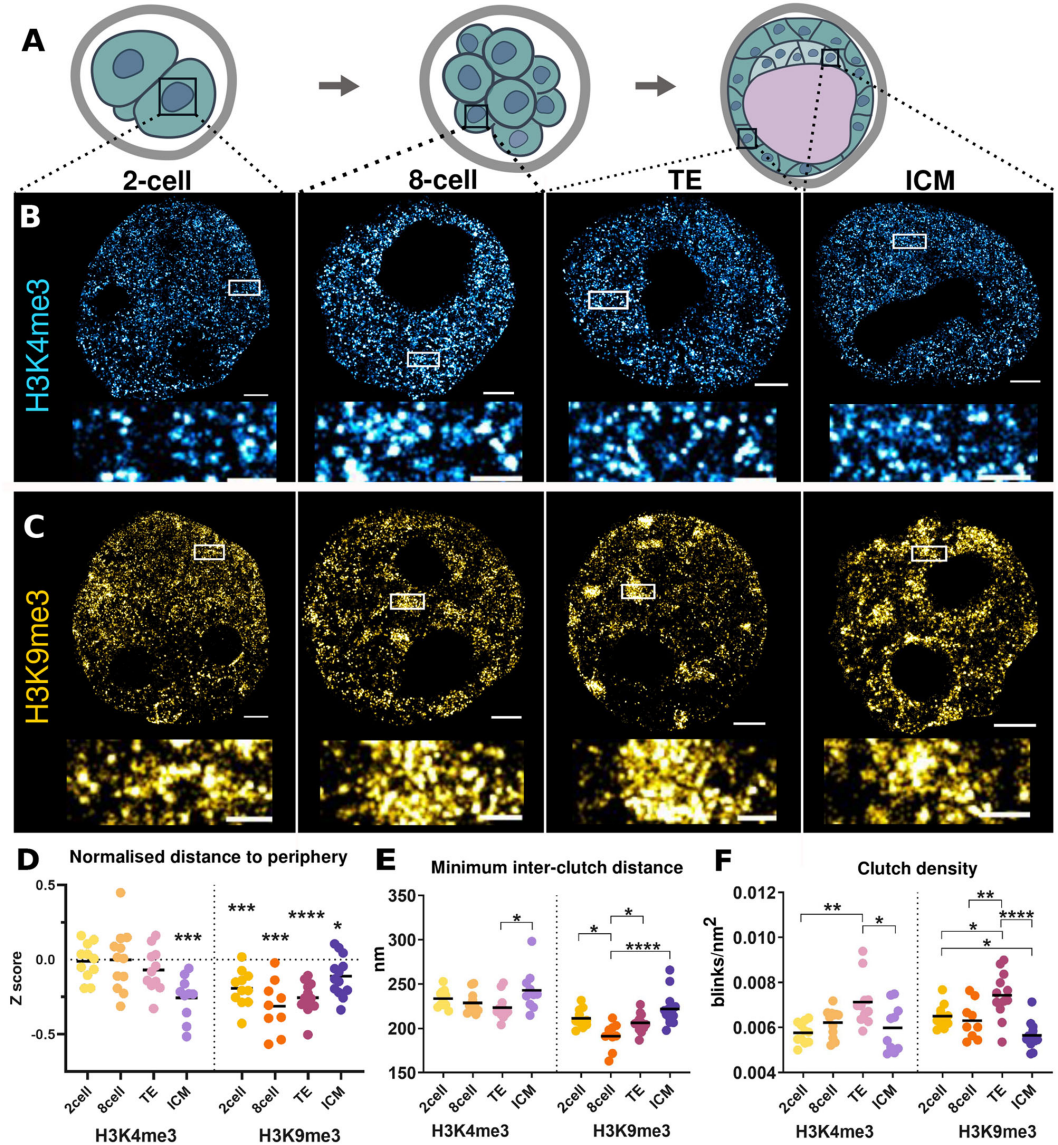
**Chromatin dynamics during preimplantation development.** (A) Schematic representation of the mouse preimplantation stages analysed. (B,C) Representative *d*STORM images of H3K4me3 (B) and H3K9me3 (C) distribution in two-cell, eight-cell, and blastocyst ICM and TE nuclei. Magnification of insets are shown below the images. Scale bar: 2 µm (B,C), 500 nm (insets). (D) Normalised distance of blinks to the nuclear periphery, where 0 is the mean distance of every possible spot in the nucleus to the nuclear periphery. Positive values indicate that the signal is farther from the nuclear lamina, and negative values closer. *P*-values for H3K4me3: 0.0004 (ICM); HK9me3: 0.0004 (two-cell), 0.0008 (eight-cell), <0.0001 (TE), 0.0113 (ICM). (E) Proximity between clutches. *P*-values for H3K4me3: 0.00287 (TE versus ICM); H3K9me3: 0.0167 (two- versus eight-cell), 0.0443 (eight-cell versus TE), <0,0001 (eight-cell versus ICM). (F) Clutch density calculated as the number of blinks divided by nuclear area of the clutch. *P*-values for H3K4me3: 0.0023 (two-cell versus TE), 0.0447 (eight-cell versus TE), 0.0161 (TE versus ICM); H3K9me3: 0.0277 (two-cell versus TE), 0.0466 (two-cell versus ICM), 0.0092 (eight-cell versus TE), <0.0001 (TE versus ICM). On the graphs (D-F), *P*-values are represented as follows: *, *P*-value<0.01; **, *P*-value<0.001; ***, *P*-value<0.0001; ****, *P*-value<0.00001. *P*-values were determined by One sample *t*-test (D) and by Tukey test for multiple comparisons (E and F).

We followed the temporal dynamics of chromatin organisation during the first stages of mouse development by imaging H3K4me3 and H3K9me3 histone marks at the early two-cell stage, eight-cell stage, and in E3.5 blastocysts (Fig. 2A–C and Fig. S2A,B). In order to distinguish ICM and TE cells in the blastocyst, we carried out immunochemistry followed by confocal microscopy for SOX2 and CDX2 together with that for histone modifications (Fig. S2C,D). These markers show no overlap whatsoever at this stage (Wicklow et al., 2014) and allow to unambiguously distinguish the two cell types (SOX2 for ICM, and CDX2 for the TE).

We first focused on studying the distribution of chromatin labelled for the two different histone marks within the nuclei. To do so, we calculated the distance to the nuclear periphery for each *d*STORM blink and normalised it with the average distance of all possible locations inside the nucleus (Fig. S3A–C), where positive or negative values correspond to blinks localised distant or close to nuclear periphery respectively. A value of zero corresponds to an equal distance to the periphery and centre of the nucleus. We observed that euchromatin (labelled with H3K4me3) shows a homogenous distribution throughout the nucleus at all stages analysed (Fig. 2B and Fig. S2A). The quantification of the images confirmed that at the two- and eight-cell stages the blinks are equally distributed among the periphery and the nuclei centre (Fig. 2D). However, at the blastocyst stage, while H3K4me3 labelled euchromatin is also homogenously distribute in TE nuclei, it is closer to the periphery in the case of ICM nuclei, as shown by a significantly negative value for normalised distance (Fig. 2D). In the other hand, heterochromatin (labelled with H3K9me3) is enriched at the perinuclear regions at two- and eight-cell stages, and in TE nuclei (Fig. 2C and Fig. S2B), with and increasing displacement towards the periphery along development (as evidenced by increasing negative values for normalised distance as shown in Fig. 2D). In contrast, we observed no significant enrichment of heterochromatin close to the nuclear lamina in ICM blastocyst nuclei (Fig. 2D).

Next, we studied the proximity between nucleosome clutches labelled with either mark by quantifying the distance between a clutch and its nearest neighbour. We found that the distance between H3K4me3 clutches remains stable along development. In contrast, nucleosomal clutches in ICM cells are significantly more isolated that in TE cells (Fig. 2E; Fig. S3D). As for heterochromatin clutches labelled by H3K9me3, overall, they are closer together than H3K4me3 labelled clutches, markedly at the eight-cell stage. Again, ICM nuclei show a more dispersed pattern as compared not only to TE nuclei but also to earlier stages (Fig. 2E; Fig. S3E). A similar conclusion was reached when measuring clutch density. There is an increase for both histone marks along development, with the exception of ICM nuclei, that show a significantly lower density for clutches labelled with H3K4me3 and H3K9me3 (Fig. 2F; Fig. S3F,G).

In summary, our data suggest that while H3K4me3 labelled euchromatin is mostly found in the central region of the nucleus, H3K9me3 heterochromatin is significantly associated with the nuclear periphery. Nucleosome clutches decrease the distance between them along development and, in a complementary fashion, show higher density at later developmental stages. This tendency is more obvious for H3K9me3 than for H3k4me3 nucleosome clutches. In addition, our data show that there is a clear difference in chromatin organisation between the two distinct cell types found in the blastocyst, as nucleosome clutches (both in eu- and heterochromatin) are closer and at higher densities in TE than in ICM nuclei.

### Higher-order chromatin folding along mouse preimplantation development

**Fig. 3.**
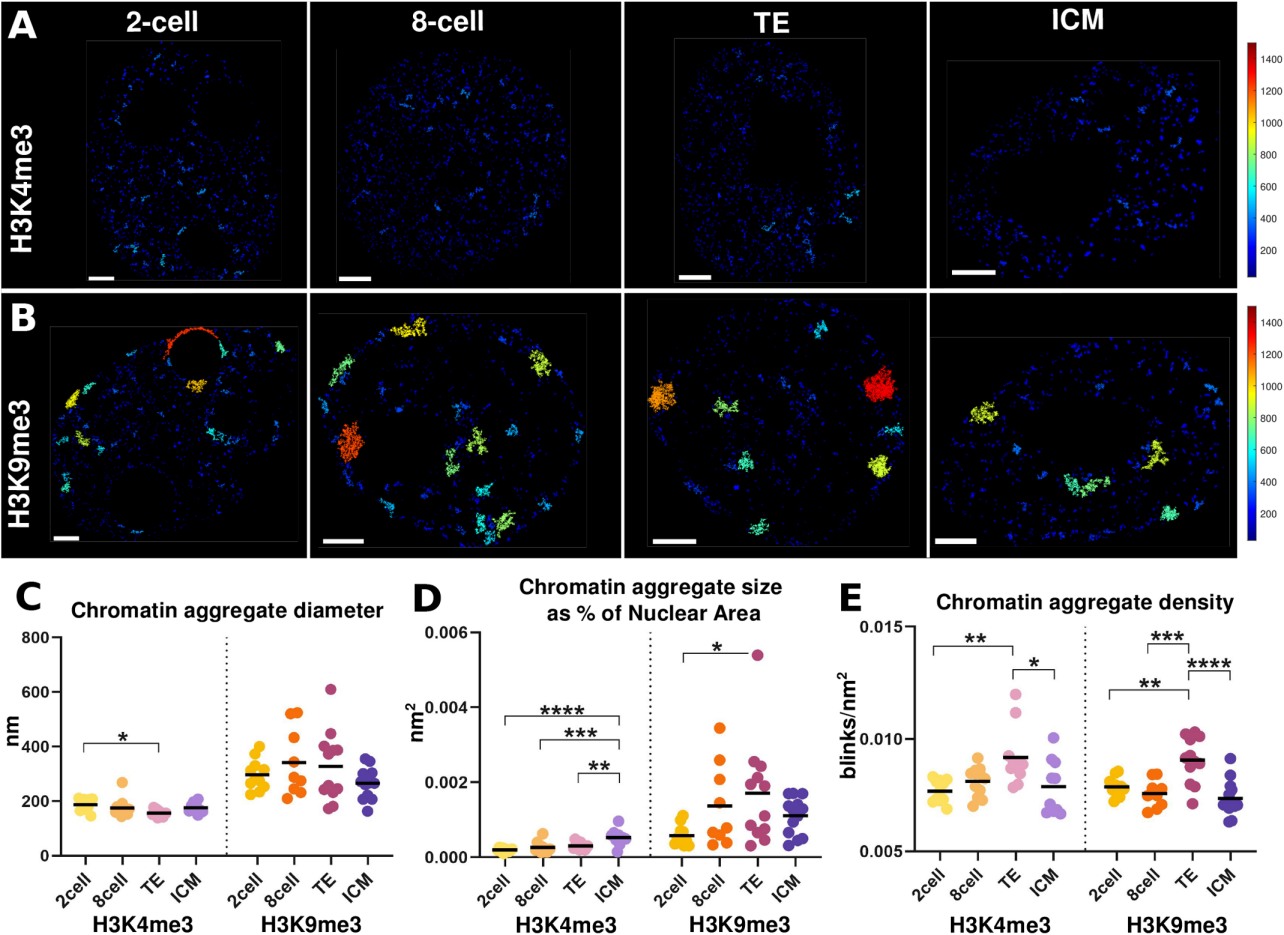
**Chromatin aggregates along preimplantation development.** (A,B) Representative images of H3K4me3 (A) and H3K9me3 (B) localisation in two-cell, eight-cell, ICM and TE nuclei, coloured by the diameter of the chromatin aggregates analysed. (C) Diameter of the chromatin aggregates. *P*-value for H3K4me3: 0.0132 (two-cell versus TE). (D) Size of the chromatin aggregates relative to the total nuclear area. *P*-values for H3K4me3: <0.0001 (two-cell versus ICM), 0.0002 (eight-cell versus ICM), 0.0027 (TE versus ICM); H3K9me3: 0.0275 (two-cell versus TE). (E) Chromatin aggregate density. *P*-values for H3K4me3: 0.0038 (two-cell versus TE), 0.0173 (TE versus ICM); H3K9me3: 0.0027 (two-cell versus TE), <0.0001 (TE versus ICM), 0.0003 (eight-cell versus TE). On the graphs (C-E), *P*-values are represented as follows: *, *P*-value<0.01; **, *P*-value<0.001; ***, *P*-value<0.0001; ****, *P*-value<0.00001. *P*-values were determined by the Tukey test for multiple comparisons.

We next analysed the characteristics of chromatin aggregates, obtained following the process of hierarchical segmentation previously described (Fig. 1H). Visual inspection of the results of the segmentation process already showed that H3K9me3 labelled heterochromatin forms much larger clusters than H3K4me3 labelled euchromatin, throughout all stages of development we analysed (Fig. 3A,B). Interestingly, while ICM nuclei also form large heterochromatin aggregates, we observed that their size was not as large as in TE nuclei from equal stage blastocyst or even at earlier stages (note the lack of red-coloured aggregates in the representative ICM nuclei shown in Fig. 3B). Quantitative analysis of the images showed that most of the H3K9me3 labelled chromatin was found in large chromatin aggregates in both eight-cell stage embryos and TE cells (Fig. S4A,B), suggesting that at these stages, heterochromatin is preferentially localised in larger structures than in intermediate or dispersed aggregates.

To examine whether the dynamics of these larger chromatin structures changed along early development, we compared their average diameter along development. We found that for H3K9me3 labelled chromatin there were no significant differences between different developmental stages and cell types (Fig. 3C). Their average diameter was over ∼310 nm, significantly larger than H3K4me3 labelled aggregates at ∼170 nm, and in agreement with our prior observation (Fig. 3A,B). We obtained similar results if we measured instead the area of the largest chromatin aggregates (Fig. S4C). We next examined if the amount of chromatin included in aggregates changed over developmental time by quantifying the proportion of the nuclear area occupied by these aggregates. In this way, we take into account the progressive reduction in the size of the nucleus that occurs during these stages (Fig. S4D). We observed a small but significant increase over time in the ratio aggregate/nucleus for H3K4me3, which reached a maximum in the ICM (Fig. 3D). Despite a higher heterogeneity, this trend was also observed for H3K9me3 labelled heterochromatin (Fig. 3D). Finally, we measured the density of chromatin aggregates, measured as the number of blinks per area, and observed a higher density in TE nuclei of both H3K4me3 and H3K9me3 labelled chromatin aggregates as compared to the ICM, as well as other stages (Fig. 3E). These results show that during development, while the nucleus decreases its size, the region of heterochromatin dense regions remains mainly unaltered, occupying a larger percentage of the nuclear area. In addition, we observe a recruitment of chromatin to these H3K9me3 labelled areas and their compaction in the TE.

## DISCUSSION

We have optimised *d*STORM-based super-resolution imaging for micrometric spherical tissues. This has allowed us to resolve to the scale of nanometres, the organisation of euchromatin or heterochromatin, labelled with antibodies for H3K4me3 and H3K9me3 respectively, in whole-mount preimplantation mouse embryos.

Previous biochemical studies have shown that, after the two-cell stage, the pattern of H3K9me3 and H3K4me3 remains mainly unchanged during mouse preimplantation development (Wang et al., 2018; Zhang et al., 2016). Our work provides evidence for changes in the distribution of heterochromatin in the nucleus at these stages, with marked differences between the first two different cell types to appear in the embryo, the ICM and the TE.

We observed clear contrasts between H3K4me3 labelled euchromatin and H3K9me3 labelled heterochromatin at all stages examined. H3K4me3 domains are smaller than H3K9me3 domains, and show a more even distribution in the nucleus. On the contrary, H3K9me3 labelled chromatin is organised in more heterogeneous domains, including large aggregates, which tend to localise closer to the nuclear lamina.

While H3K4me3 labelled clutches and aggregates are relatively stable at the stages we examined, H3K9me3-labelled heterochromatin shows a more dynamic behaviour, getting nearer to the periphery or among clutches from the two-cell to the eight-cell and the blastocyst TE nuclei. However, we consistently observed a discordant behaviour of blastocyst ICM nuclei, for these and other measurements such as clutch density, and for both H3K4me3 and H3K9me3 labelling. These observations support the idea that there are different histone mobility rates and chromatin compaction levels between pluripotent and lineage-restricted cells (Ahmed et al., 2010; Bošković et al., 2014; Gómez-García et al., 2021).

Overall, we do not observe that less committed cells (toti- or pluripotent) of earlier stage embryos have a more open chromatin state, which would gradually compact as differentiation takes place (e.g. to the trophectoderm). However, the ICM changes the state of its heterochromatin to a less compacted and specified state that does not necessarily correspond to earlier totipotent stages of development. In this regard, we can consider that pluripotency-associated chromatin states are a novel, actively acquired condition in the embryo, and that there is not a passive maintenance of earlier chromatin states. These observations would be in agreement with recent studies in tissue culture that have shown a less compacted heterochromatin in pluripotent cells (Xu et al., 2018), processes of de-compaction in cellular transformation during early carcinogenesis (Xu et al., 2020) and an increase in compaction and in neural differentiation (Gómez-García et al., 2021).

The mayor differences we observe are between H3K9me3 labelled chromatin in blastocyst TE and ICM nuclei. This is not completely unexpected, as it coincides with the more advanced differentiation state of the already lineage-restricted TE cells compared to pluripotent ICM cells. This is the first lineage specification event taking place during mammalian development, and molecular differences between cells start to appear as early as the eight- to 16-cell stage (Frum and Ralston, 2015; Menchero et al., 2019). However, we did not observe any bi-modal distribution of chromatin properties in our analysis at the eight-cell stage, that could anticipate the determination of the two different cell types in the blastocysts. By this time, certain components of the signalling pathways involved in TE specification already show differential distribution in the embryo (Hirate et al., 2012; Menchero et al., 2019), sign of the first differentiation event to take place. Therefore, this suggests chromatin changes in the early embryo would be downstream of transcription factor driven lineage specification.

Finally, while the study of chromatin organisation in single-cells by genome-wide biochemical assays has been recently applied to the early mammalian embryo (Collombet et al., 2020; Xue et al., 2013; Guo et al., 2017; Zhu et al., 2018; Lu et al., 2016; Ke et al., 2017; Du et al., 2017; Zhang et al., 2016), our work offers a complementary tool to study this issue. The combination of the approach described here with other labels, such as for chromatin bound factors or single genomic loci, can provide the opportunity to achieve a more complete understanding of the temporal and spatial dynamics of how chromatin is structured at the very beginning of development.

## MATERIALS AND METHODS

### Embryo collection and immunofluorescence

CD1 mouse embryos were collected from superovulated females at embryonic stages E1.0 (32–36 h after injection of hpHCG in order to avoid the imaging of G2 nuclei that would have already duplicated their genome and therefore carry double amount of histones; Moore et al., 1996; Nagy et al., 2003), E2.5 and E3.5 by flushing the oviduct or the uterus with M2 medium (M7167, Sigma-Aldrich) and were fixed for 10 min (min) in 4% PFA in PBS 1X.

After fixation the embryos were permeabilised with 0.5% Triton X-100 in PBS (PBST 0,5X) for 25 min, washed once in 0.1% Triton X-100 in PBS (PBST 0,1X) and blocked for 1 h in 10% of FBS in PBST 0,1X at room temperature. Embryos were incubated at 4°C overnight with primary antibodies diluted to optimised concentrations: rabbit polyclonal α-H3K4me3 [1:500 and 1:300] (ab8580, Abcam; batch number GR45436-1), rabbit polyclonal α-H3K9me3 [1:500 and 1:300] (ab8898, Abcam; batch number GR336562-1), mouse monoclonal α-CDX2 [1:200] (MU-392A-UC, Biogenex; batch number MU392A1107), goat polyclonal α-SOX2 [1:100] (AF2018, R&D systems; batch number KOY0316101). After a 30 min wash in PBST 0,1X, staining with secondary antibodies was performed for 1 h in the dark. Secondary Alexa Fluor 647-conjugated chicken α-rabbit and goat α-mouse, 488-conjugated donkey α-mouse and 568 donkey α-goat antibodies (Life Technologies) were used at a 1:500 dilution. Finally, the embryos were washed for 30 min in PBST 0,1X and for 10 min in PBS 1X.

CD1 mice (Charles Rivers) were housed and maintained in the animal facility at the Centro Nacional de Investigaciones Cardiovasculares (Madrid, Spain) in accordance with national and European legislation. Procedures were approved by the CNIC Animal Welfare Ethics Committee and by the Area of Animal Protection of the Regional Government of Madrid (ref. PROEX 196/14).

### Mounting and sample preparation

After trying different methods to overcome the physical (embryo volume and focus drifts), technical (working distance and depth of field of the microscope) and physicochemical (need for a reductive environment for a good blinking of the fluorophores) challenges, we established the following protocol and mounting media for our samples. Embryos were individually plated in drops of PBS 1X in 1.5H glass-bottomed dishes (81158, Ibidi). Excess of liquid was let to evaporate and the dish was covered with a buffer containing β-Mercaptoethylamine (MEA) (30070, Sigma-Aldrich) at 30 mM and pH8.3, diluted in 99.5% Glycerol (24388.295, VWR Chemicals) to avoid the drift during image acquisition. The glycerol containing buffer did not affect blinking, as shown by Goossen-Schmidt et al. (2020). To avoid oxygen diffusion and maintain a reductive environment, a 25 mm round coverslip (64-0715, Warner Instruments) was placed over the preparation and sealed with nail polish.

### Data acquisition

Images of two-cell, eight-cell, ICM and TE were acquired on a Leica GSD (Ground-State Depletion) super-resolution system (Leica Microsystems GmbH, Germany) equipped with a 160× oil immersion objective numerical aperture 1.43, and an EMCCD back-illuminated ANDOR iXON Ultra DU897 camera. We used a 642 nm laser for imaging (pumping and normal excitation cycle), and a 405 nm laser for back-pumping. For E3.5 stage embryos, 532 nm and 488 nm lasers were briefly used to differentiate ICM cells from TE cells by differential CDX2 and SOX2 expression as detected by antibody staining. Total number of nuclei imaged, as well as the number of embryos and independent litters used are available in Table S1.

Oblique illumination was applied to obtain a focal plane of 130 nm–150 nm thickness. Between 35,000 to 40,000 frames were recorded per image with a 93 EM gain and 8.95 ms exposure. The focal plane was selected depending on two conditions: the proximity to the objective and the maximum diameter of the nucleus to analyse.

### Data pre-processing

To generate the coordinate-map from dSTORM videos we used ThunderSTORM v1.3 (Ovesný et al., 2014) for ImageJ v1.52e software (Schindelin et al., 2012). First, drift was corrected through cross correlation. Next, the localisations were filtered setting the intensity threshold at a range from 300 to 5000 photons and the uncertainty and the sigma were set at 35 nm and 200 nm respectively.

To avoid any interference of the extranuclear area in the analysis, the nucleus was manually segmented from reconstructed images with 16 nm/pixel. Blinks with coordinates outside the nucleus were discarded from the coordinates-map for further analyses.

### Density-based homogenisation of *d*STORM data

To prevent the large variability of blink densities from hindering proper analysis and comparison between samples, a density-based homogenisation strategy was used to obtain coordinate-maps with similar density of blinks for all samples. A global density of blinks was fixed at 0.001 blinks/nm^2^. Only blinks from the last k frames of a *d*STORM video were used, where k was selected to better approximate the target global density of blinks in the corresponding segmented area. Samples with low density that failed to approach the target density were discarded. In this way, we avoided potential instabilities occurring during the first frames of the acquisition, what also allowed us to use density-based clustering algorithms to determine spatial groupings of blinks in an unbiased manner.

### Clustering

Clustering of blinks was performed with a hierarchical strategy: blinks were grouped in clutches, and clutches were grouped in chromatin aggregates. First, DBSCAN (Ester et al., 1996) unbiased clustering was used to clean noisy blinks by detecting outliers (neighbourhood search radius, ε=60 nm; minimum number of neighbour's required for core point selections, minimum points=10). Next, remaining blinks were grouped into clutches using hierarchical clustering, setting a cut-off for maximum distance between blinks within a cluster (maximum intracluster distance, maximum distance=160 nm; and minimum number of blinks to define a clutch, minimum blinks=10). Clutches with low densities (less than 5× global density) were filtered out. Finally, DBSCAN grouped clutches into chromatin aggregates based on minimum distances between their blinks (ε=30 nm, minimum points=1). All parameters and cut-offs were selected based on previous work (Fang et al., 2018; Otterstrom et al., 2019; Ricci et al., 2015; Xu et al., 2018; Nozaki et al., 2017; Leterrier et al., 2015) and after visual examination of results.

### Extraction of measurements

Distance to nuclear periphery was computed analysing blinks, subtracting the average distance of all possible locations and dividing this value by the standard deviation. This generates a z-score that reflects normalised distances values of positions within the nucleus, distant (>0) or closer (<0) to the periphery.

The areas of higher-level structures (clutches and chromatin aggregates) were calculated from the determination of the boundaries defined by their contained blinks (not forcing a convex shape), allowing the computation of the percentage of occupation with respect to the total nuclear area, an approximate diameter and the density of blinks in the structure. Proximity between clutches was calculated as the average distance (measured from their boundaries) between a structure and its 10 nearest neighbours.

Measurements per image were obtained by averaging the structures in the sample or by weighting those values by the number of blinks in the clustered structure. Specifically, we extracted and compared: a) mean normalised distance from blinks to periphery, b) mean density of blinks in pre-filtered clutches, c) mean proximity between clutches, d) weighted mean of approximate diameter of chromatin aggregates, e) weighted mean of percentage of area occupied by chromatin aggregates, and f) weighted mean density of blinks in chromatin aggregates.

### Calculation of radial distribution (RDF) and H functions

Both RDF and H functions provide information about the degree of particle clustering from blinks. The RDF measures how the density of particles varies as a function of distance from a reference particle, by quantifying the probability of finding particles within a torus with radii r1 and r2 from another reference particle (Cohn, 1968). For regular sized and spaced particle clusters, the mean cluster radius and the separation between them can be estimated from the width of the first density peak and the separation between subsequent peaks, respectively, in RDF. Unclustered data display an RDF of around 1 for all distance values.

The H-function is a derivation of Ripley's K function (Ripley, 1977; Kiskowski et al., 2009) that provides a measurement of the average amount of molecules that lie at distance ‘r’ from a reference molecule, divided by the mean global density and the total number of molecules. The H-function normalises these values to area and radius, conforming a function that provides information about the radii at which the density is higher than the global density. Unclustered data display an H-function of around 0 for all distance values. For regular sized and spaced particle clusters, the mean cluster radius, the proportion of clustered particles, and the separation between them can be estimated from the distance at which the density peak is reached, its height, and the distance at which the function crosses the X-axis, respectively.

However, the heterogeneity observed in molecular patterns of biological samples hampers the proper interpretation of RDF and H functions, affecting the density peaks and shapes of these functions. In any case, qualitative comparisons of these functions show differences in the degree of particle clustering and heterogeneity of cluster sizes between different conditions.

Computation of these functions was performed using the blink coordinate-maps after density-based homogenisation of the whole image, studying a range from 0 to 500 nm with steps of 10 nm. The implementation presented in the published software MIiSR (Caetano et al., 2015) was adapted to improve the precision of results by including a more sophisticated border correction. Instead of selecting as reference blinks for computation only those ones located farther than the maximum distance tested (500 nm) from the border of the rectangular region of interest (ROI), we used the actual boundary of the nucleus to that end. Furthermore, we adapted the selection of reference blinks to disregard only the ones with distance to the edges lower than the tested distance in each case. This results in a more precise output where a higher number of blinks are used for computation.

### Calculation of F-function

Another way to detect irregular spatial distributions of particles is using the F-function: the cumulative distribution of the distance from arbitrary nuclear positions to the nearest particles (Diggle, 2003). F(r) represents the nuclear volume fraction that is at a distance less than ‘r’ from the particles in the pattern, revealing trends for clustering (large, aggregated patterns) or regularity (small regular patterns). Again, the natural heterogeneity of biological samples limits the interpretation and applicability of F-function when a mixture of different levels of aggregation of particles are present. However, it still can be useful to identify samples with bigger empty areas (where no particles are present).

F-functions were computed to study spatial distributions of clutches within the nuclei. We used ImageJ and ‘spatial statistics’ plugin (Diggle, 2003; Andrey et al., 2010) to analyse labelled images of clutch-centres reconstructed at 16 nm/pixel resolution. A stochastic strategy was used to randomly generate the set of arbitrary positions within the nucleus (evaluation points). The cumulative F-function was estimated using the distances from each evaluation point to its closest clutch-centre.

Due to the arbitrary shape of the nucleus, deviation from spatial randomness can only be quantified by comparing the F-function of the observed clutch distribution with a completely random one. A Monte-Carlo approach was followed to randomly shuffle the pattern of clutch-centres in order to obtain a reference cumulative function. We used 10.000 evaluation points and 25 randomly generated clutch patterns to estimate the F-functions. A final value of ‘deviation from randomness’ was obtained by computing the area between functions for observed and random clutch patterns.

### Statistical analysis

Statistical analyses were performed with GraphPad Prism v.8.4.3 (GraphPad Software, USA).

### Software

Image processing, manual segmentation of nuclei, and computation of F-functions made use of ImageJ v1.52e. The analytical pipeline was implemented in Matlab R2020a (The Mathworks, USA). Graphics were generated with Matlab and GraphPad Prism.

## Supplementary Material

Supplementary information
