# Supplementary material for: Chromatin dynamics through mouse preimplantation development revealed by single molecule localisation microscopy
Source: Biol Open. 2022 Jul 29;11(8):bio059401. doi: 10.1242/bio.059401 (PMC9346283; doi:10.1242/bio.059401)
Supplement: Supplementary information [file biolopen-11-059401-s1.pdf]

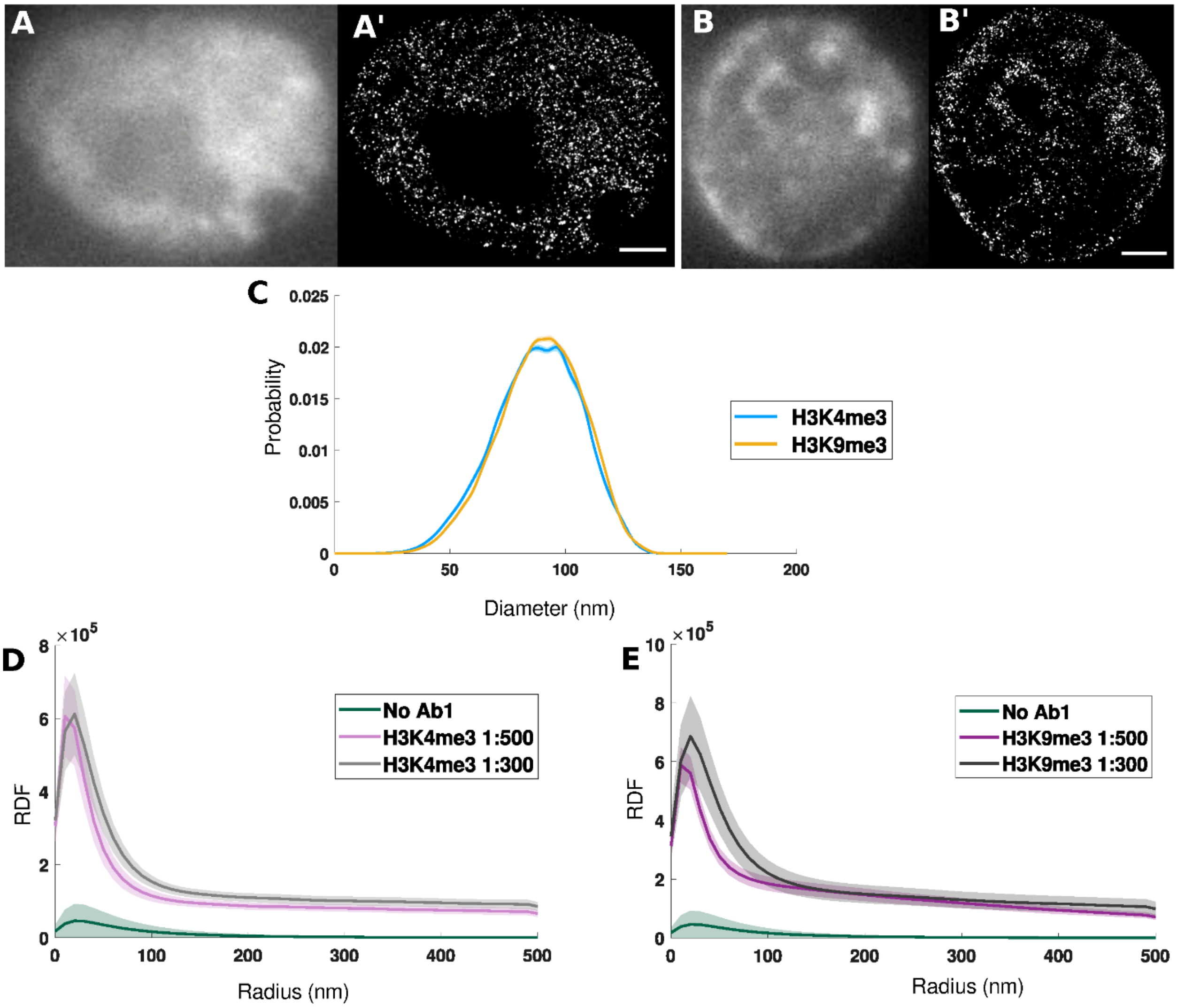

Fig. S1. *d*STORM enhances resolution of chromatin imaging in mouse preimplantation embryos.

Oblique illuminati++on (**A**, **B**) and *d*STORM images (**A'**, **B'**) of H3K4me3 (**A**, **A'**) and H3K9me3 (**B**, **B'**) stained TE nuclei. Scale bars: 2 µm. **C**, Probability density function displaying the distribution of blinks in clutch sizes. **D** and **E**, RDF (Radial Distribution Function) of H3K4me3 (**D**) and H3K9me3 (**E**) at 1:500 and 1:300 antibody concentrations, where the functions show the variation of the density of particles in relation to the distance from a reference particle.

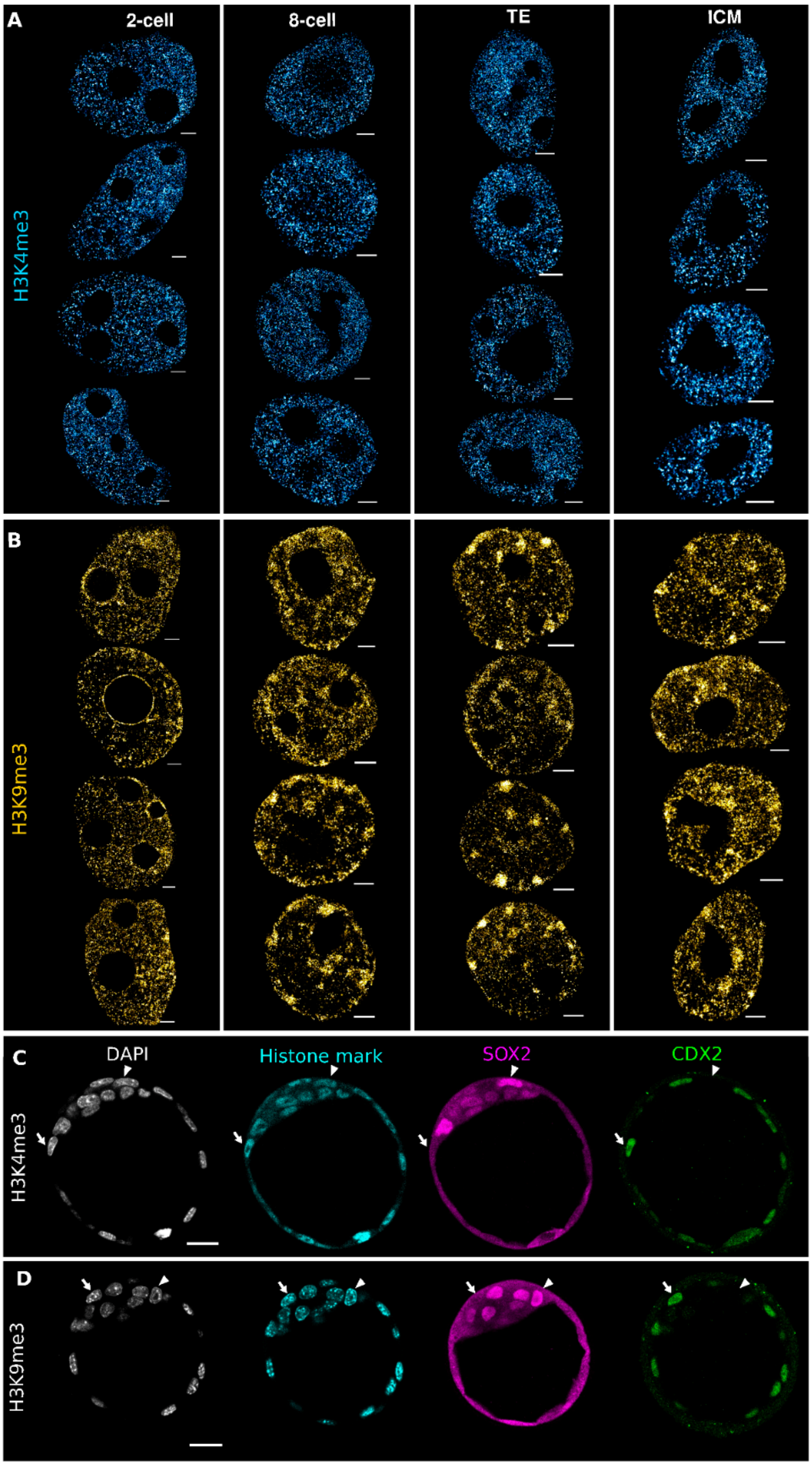

Fig. S2. *d*STORM images of epigenetic marks at different developmental stages.

Representative *d*STORM images of several nuclei stained for H3K4me3 (**A**) and H3K9me3 (**B**) at 2 cell, 8 cell, and TE and ICM nuclei from the blastocyst stage. **C** and **D**, Confocal images of representative blastocysts stained for H3K4me3 (**C**) and H3K9me3 (**D**) histone marks (cyan), SOX2 (magenta) and CDX2 (green). Nuclei are stained with DAPI (grey). Arrowheads indicate SOX2+/CDX2- ICM nuclei, and arrows SOX2-/CDX2+TE nuclei. Scale bars: 2 μm.

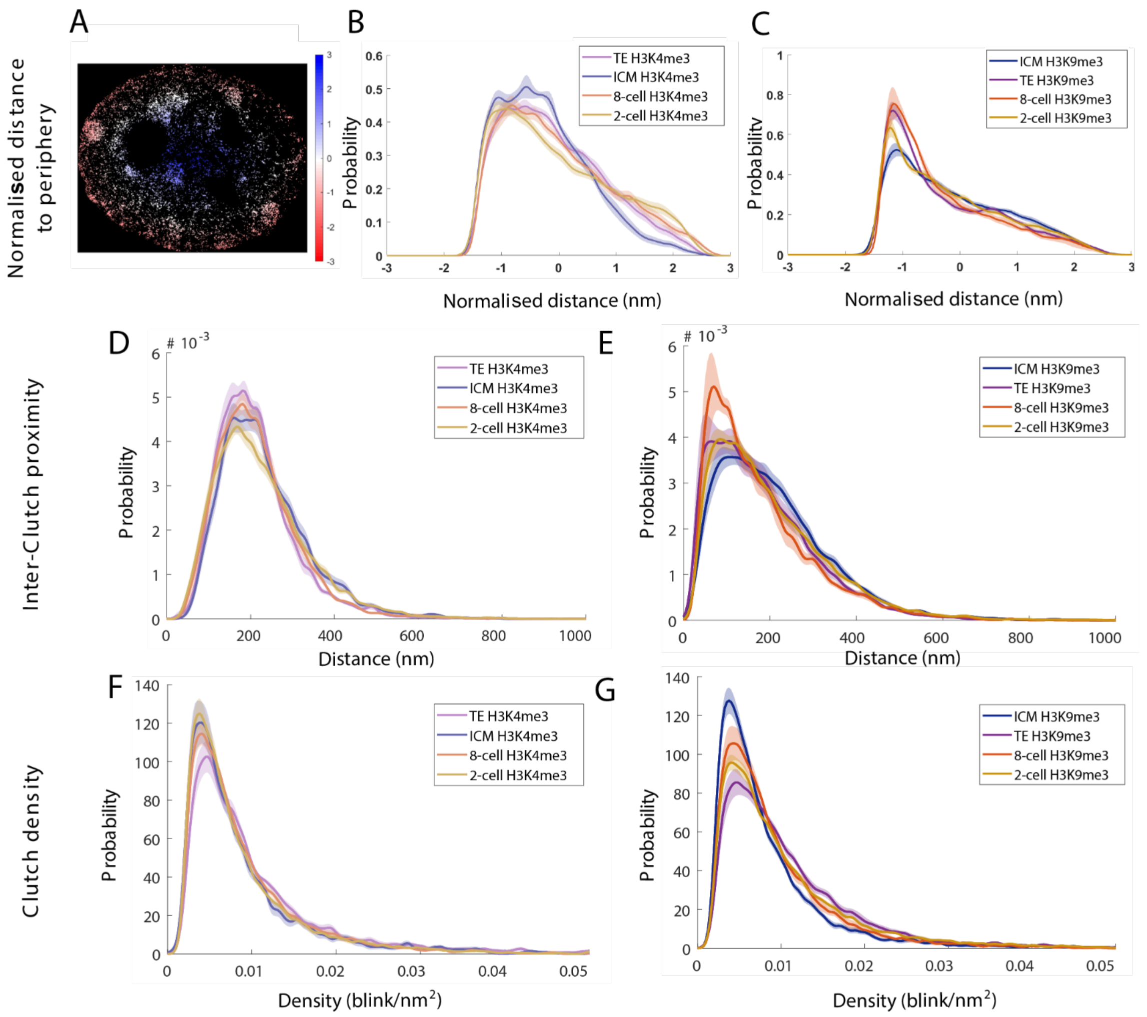

Fig. S3. Dynamics of nucleosome clutches in preimplantation development.

A Representative dSTORM image of H3K4me3 immunohistochemistry signal of an 8-cell stage nuclei colored by the distance to the nuclear periphery. **B** and **C**, Probability density functions showing the distribution of blink distances to the nuclear periphery for H3K4me3 (**B**) or H3K9me3 (**C**). **D** and **E**, Probability density functions of proximity between H3K4me3 (**D**) and H3K9me3 (**E**) clutches. **F** and **G**, Probability density functions of the density of clutches measured as number of blinks divided by the clutch area for H3K4me3 (**F**) and H3K9me3 (**G**).

Biology Open • Supplementary information

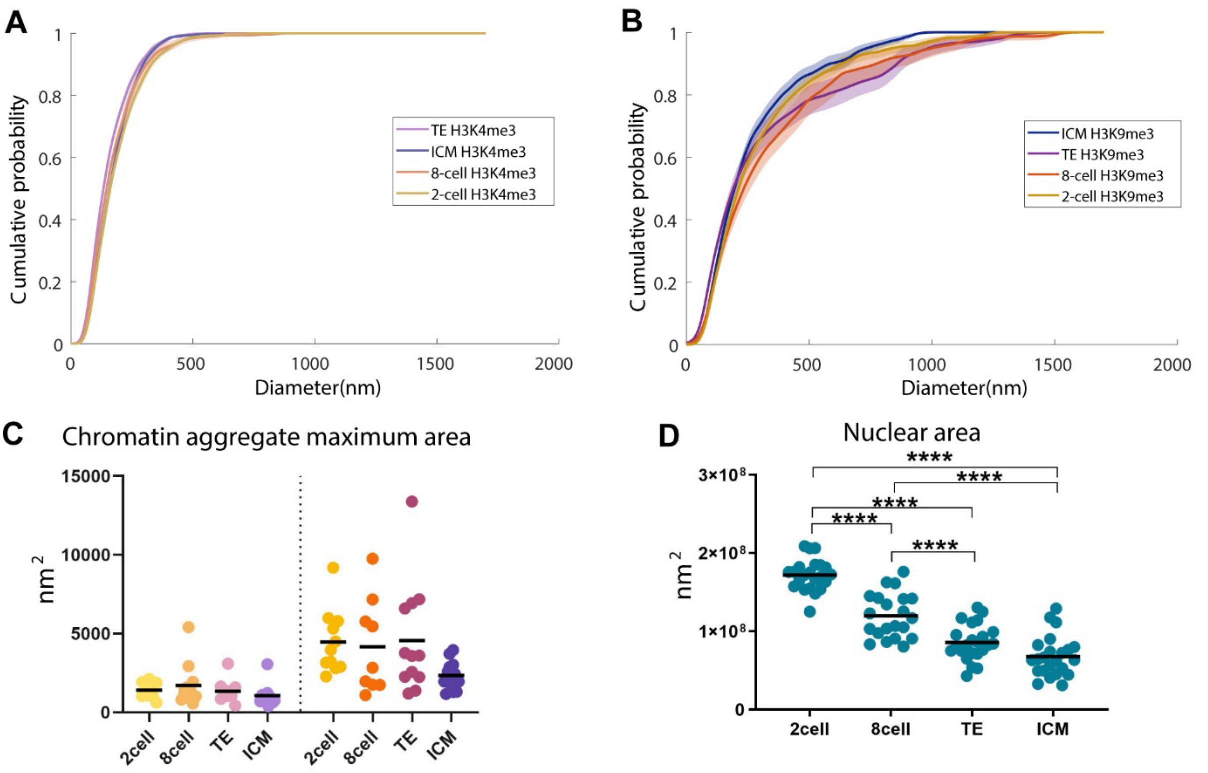

Fig. S4. Chromatin aggregates along preimplantation development.

A and **B**, Cumulative distribution function displaying the percentage of blinks included in chromatin aggregates up to a specific size for H3K4me3 (**A**) or H3K9me3 (**B**). **C**, Area of the largest chromatin aggregate stained for H3K4me3 and H3K9me3. **D**, Change in nuclear area along preimplantation development. ****, all p-values are < 0,00001; as determined by the Tukey test for multiple comparisons.

Table S1. detailed information on the number of independent cells imaged in this study

stage	label	nº of cells	nº of embryos	nº of litters
2cell	H3K9me3	11	11	2
2cell	H3K4me3	11	11	2
8cell	H3K9me3	9	9	2
8cell	H3K4me3	12	11	3
ICM	H3K9me3	13	12	4
ICM	H3K4me3	10	7	4
TE	H3K9me3	12	11	5
TE	H3K4me3	11	10	3
